# Automated Droplet-Based Microfluidic Analyzer for *In Situ* Monitoring of Ammonium Ions in River Water

**DOI:** 10.1021/acsestwater.4c01231

**Published:** 2025-07-14

**Authors:** Wahida T. Bhuiyan, Jelena Milinovic, Brett Warren, Yong-Qiang Liu, Adrian M. Nightingale, Xize Niu

**Affiliations:** † Faculty of Engineering and Physical Sciences, 7423 University of Southampton, Southampton, SO17 1BJ, U.K.; ‡ SouthWestSensor Ltd., 2 Venture Rd, Chilworth, Southampton SO16 7NP, U.K.

**Keywords:** Droplet microfluidics, water quality, continuous monitoring, modified Berthelot method, colorimetric ammonium detection

## Abstract

Accurate *in situ* monitoring of ammonium ion (NH_4_^+^) levels in water bodies is fundamental for assessing water quality and potential contamination. Here, we present a novel automated droplet-based microfluidic analyzer for *in situ* monitoring of NH_4_^+^ concentrations in river water. The developed analyzer miniaturizes the modified Berthelot colorimetric method into submicroliter droplets contained within oil flow. The use of droplets enables measurements at high frequency (10 s per measurement) with low reagent consumption (0.3 μL per measurement). NH_4_^+^ measurements showed linear responses up to 2 mg L^–1^, and a limit of detection (LOD) of 0.11 mg L^–1^. The system was tested with river water samples collected from the River Itchen (Southampton, UK), with excellent agreement (*p* < 0.05) between the droplet microfluidic analyzer and standard benchtop analysis. The analytical performance of this microfluidic analyzer showed that it could be applicable to sustainable *in situ* monitoring of NH_4_^+^ levels in a wide range of water bodies and scenarios, including rivers, lakes, aquaculture, and/or industrial effluents.

## Graphical abstract

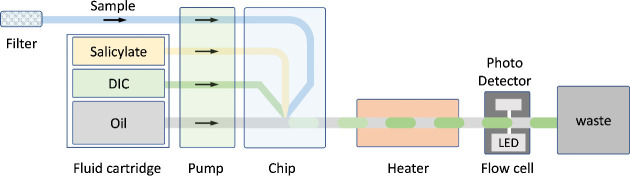

## 1 Introduction

Ammonia (NH_3_) is a key component of the nitrogen (N) cycle and is a fundamental nutrient for microbes within the aquatic ecosystem. Consequently, it is an important parameter for water quality monitoring and treatment.^1^ Dependent on temperature and pH values, ammonia can be present as protonated ammonium ions (NH_4_^+^) and deprotonated ammonia (NH_3_) with NH_4_^+^ typically predominating in natural waters.^1^

While NH_4_^+^ occurs naturally in the environment, excess quantities are often introduced by human input via wastewater outflow and poor agricultural land management. This can lead to adverse ecological effects as elevated concentrations encourage primary production and can cause algal blooms and eutrophication, which may in turn lead to hypoxia and death of aquatic fauna.^2,3^ Therefore, monitoring of NH_4_^+^ in natural waters is important to be able to detect and remediate excessive NH_4_^+^ concentrations.

The logistical problem of taking and transporting spot samples for lab analysis has led to the development of a range of different sensing technologies for *in situ* monitoring of NH_4_^+^ levels.^1,4^ Electrochemical sensors, such as ion-selective electrodes (ISEs) are sensitive, easy to fabricate, and can be miniaturized with low cost in mass production.^5^ Electrochemical sensors are susceptible to surface fouling and chemical interferences, however, and require frequent recalibration.^6^ Wet chemistry sensors take a different approach, autonomously taking samples and performing miniaturized versions of the gold-standard benchtop assay methods. As they employ well-understood assays, they offer highly selective and accurate measurements.^4^

A disadvantage of wet chemical sensors, however, is that they need to be deployed with consumables (reagents and standards) that need to be periodically replenished. Consequently, there has been a drive to develop wet chemical sensors based on microfluidics that have low internal volumes and hence reduce the amount of fluid used.^7–9^ In particular, droplet microfluidic systems, where samples are compartmentalized into small droplets (fL to μL) by the introduction of an immiscible oil^10^ have been shown to be exceptionally frugal, with a previously reported droplet-based nitrate sensor giving orders-of-magnitude reduction in fluid use.^11^ Droplet flow can also offer reduced reaction times due to enhanced mixing and, as droplet contents are encapsulated away from channel walls, surface contamination and sample smearing (via Taylor dispersion) is prevented.^10,12–15^ Compared to traditional methods, droplet-based microfluidic systems are an ideal tool for wet chemical sensing where low maintenance, high frequency measurement, and low reagent consumption are required.

Here, we report a droplet-microfluidic-based analyzer suitable for continuous measurement of aqueous NH_4_^+^ levels based on colorimetric detection using the modified Berthelot method. We describe the system components, operation techniques, and insights into analytical performances through measurements of NH_4_^+^ levels in tidal river water samples. The main benefits of this innovative system, as well as some future perspectives for its improvements have been also commented.

## 2 Materials and Analytical Methods

### 2.1 Materials and Reagents

Anhydrous ammonium chloride (99.99%), sodium salicylate (>99.5%), trisodium citrate dihydrate (99%), sodium nitroprusside (>99%), sodium hydroxide (97%) and sodium dichloroisocyanurate dihydrate (≥98%) were purchased from Sigma-Aldrich or Thermo Fisher Scientific. Ultrapure deionized water (18.2 MΩ cm^–1^, Milli-Q, Triple Red water technology) was used for all solution preparations and dilutions.

An ammonium chloride stock solution (1 g L^–1^) was prepared by dissolving 0.382 g of anhydrous ammonium chloride (dried at 105 °C for at least 2 h) in ultrapure water and diluting to 100 mL using a volumetric flask. Salicylate reagent was prepared by dissolving 13 g of sodium salicylate, 13 g of trisodium citrate dehydrate and 9.7 mg of sodium nitroprusside in ultrapure water and diluting to 100 mL using a volumetric flask. Sodium dichloroisocyanurate reagent (DIC) was made up by dissolving 3.2 g of sodium hydroxide and 0.2 g of sodium dichloroisocyanurate dehydrate in ultrapure water. The reagents and standards were stored at 4 °C in liquid pouches made from a multilayer laminate (250 mL, DaklaPack, UK) prior to use.

### 2.2 River Water Sampling

Grab water samples were collected from five different sites along the River Itchen, a chalk stream in Southampton discharging into the English Channel. The samples (20 mL) were collected at high tide using syringes (25 mL, BD Plastipak) with filters (Biofil PES = 0.45 μm) and stored in centrifuge tubes (15 mL). The syringe and centrifuge tubes were each rinsed with sample water three times before the final sample was taken.

### 2.3 Benchtop Testing Using Modified Berthelot Method

The modified Berthelot’s reaction is based on salicylate reagent replacing the corrosive and toxic phenol in the original Berthelot assay.^16^ For benchtop testing, the standard/sample, salicylate, and DIC reagents were mixed in the proportion 10.5:1:1 (v:v:v), dried at 55 °C for 10 min, in order to allow the final green-colored ammonia-salicylate complex formation to develop more quickly and complete the desired reaction more efficiently. The concentration of the reaction product was gauged by measuring final, green-colored salicylate complex absorbance at 660 nm using a spectrophotometer (Genesys 180) and well microplate reader (BMG Labtech FLUOstar Omega). Spectrophotometric cuvette readings were used to calibrate the system with standard ammonium chloride solutions, while a microplate reader was used to analyze the river samples.

### 2.4 Droplet Microfluidic Analyzer

The design and working principle of the droplet microfluidic system is demonstrated schematically in Figure 1 and the sensor prototype in Figure 2. The water sample, salicylate and DIC reagents, and fluorocarbon oil (Fluorinert FC-40) were pumped via a stepper motor-driven peristaltic pump^7,11,17^ produced in-house (Figure S1) into a 3D printed microfluidic chip (PLA material, Ultimaker 3) where the sample and reagents first merged and were then segmented into droplets on meeting the oil (Figure S2). The pump was designed to introduce the sample, reagents, and oil into the chip with fixed volumes in controlled sequence with alternating aqueous and oil pulses, which ensured robust droplet generation. Each pulse of the aqueous fluids (sample and reagents) results in a single droplet with predefined volume and composition.^7^ The pump was set at 5.5 rpm with the resulting droplet generation frequency of 5.5 droplets per minute, as the pump was designed to produce one aqueous droplet and oil pulse per turn. The droplets exited the microfluidic chip into PTFE tubing (UT7, Adtech Ltd.) with an inner diameter of 0.7 mm. The droplets had a fixed volume (approximately 750 nL, coefficient of variance <2%) and were invariant of motor speed, viscosity or droplet constituents.^7^ Here, a 3:1:1 volumetric ratio for sample: salicylate: DIC was used to cover different ranges of NH_4_^+^ concentrations, with the volumetric ratios defined by the pump design—specifically the feature size of the pump rotor for each fluidic line.^7^

**Figure 1:**
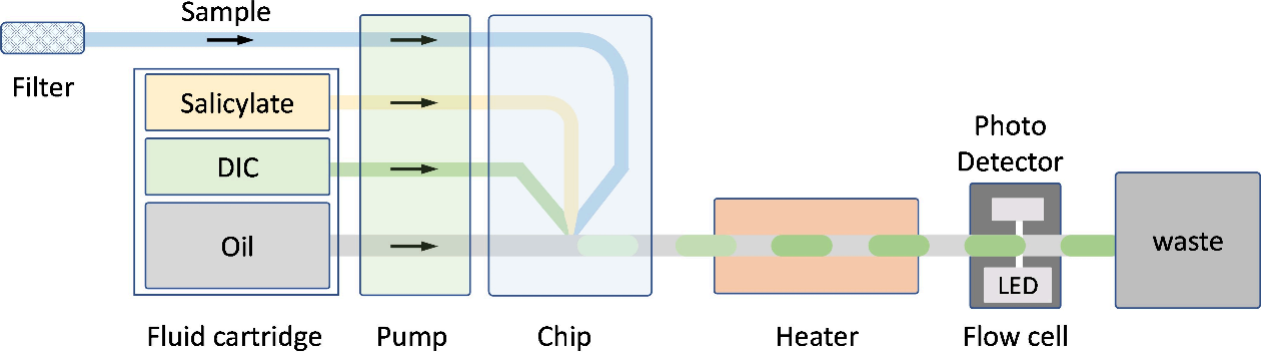
Schematic diagram of the droplet microfluidic system designed for the continuous measurement of ammonium ions in water. From left to right: the water sample (via a filter), oil and reagents (stored in fluid pouches) were pumped by a stepper motor driven peristaltic pump into a microfluidic chip to generate droplets. The droplets reacted in a heater (at 55 °C) forming the final, green-colored compound which can be detected (at 660 nm) by a downstream in-line spectrophotometer.

**Figure 2:**
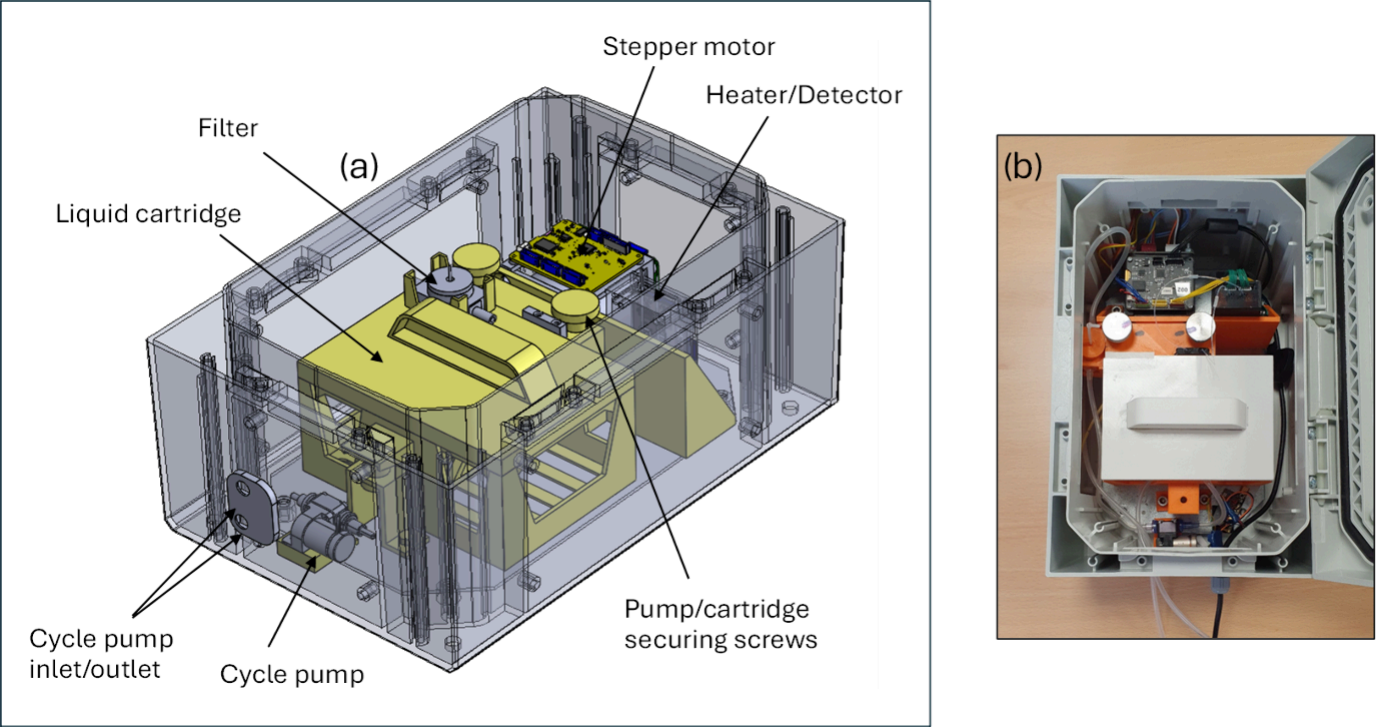
Pictures showcasing the developed ammonium ion sensor prototype. Computer aided design of the sensor showing different components, including cycle pump, heater/detector, and liquid cartridge containing the reagent pouches (a). Photograph of the sensor (b).

A heater was designed and fabricated in-house using an aluminum PCB with evenly distributed resistive heaters and an on-board thermistor.^11^ The PTFE tubing was fixed to the aluminum surface of the PCB using a 3D-printed bracket. Proportional-integral-derivative (PID) control was used to set the heater to 55 °C with a ramping time of less than 1 min from 5 °C. The retention time of the droplets in the heater was set at 2 min. After the heater, the droplets traveled through a miniaturized flow-cell for colorimetric detection at 660 nm and finally to the waste liquid pouch. The flow-cell consisted of a single 3D printed (PLA material) unit (Figure S3) with grooves for an LED (2.5 V Red LED PLCC 2 SMD, Kingbright KA-3528SRT, RS components), photodetector (TSL257-LF, RS components) and a through hole for PTFE tubing (UT7). The electronics used to control the analyzer, and the pump were designed in-house.^11^ The data were processed using a homemade graphical user interface (GUI) software.

### 2.5 Calibration of the Droplet Microfluidics System

The system was calibrated with standard samples before and after use by removing the inlet filter and connecting the inlet tube to standard solutions. The inlet of the sample tube was sequentially inserted into 500 μL vials containing standard solutions for 10 min each and then left exposed to air for 2 min between each standard to clear the system. Any liquid residue on the tube inlet was wiped away before the tube was inserted into the proceeding vial.

### 2.6 Statistical Analysis of the Results

Calibration standards and samples were analyzed in parallel using both the benchtop method and the droplet microfluidic system with respective three and ten data point replicate measurements. All results for NH_4_^+^ concentrations and standard deviations (SD) are presented in mg L^–1^ units. Linear regressions were evaluated by the coefficient of correlation (*R* ^2^). To compare results obtained by the benchtop method and droplet microfluidic system statistical analysis was performed using two-tailed Student’s *t* test at 95% significance level (*p* < 0.05). The limit of detection (LOD) for the microfluidic sensor system was calculated using the 3-sigma method.^18^

## 3 Results and Discussion

Figure 3 shows a screenshot from the GUI showing raw transmitted light measurements from the flow cell during a calibration using nine standards. The inset shows data during a four min period in more detail, with a characteristic wave profile where each trough corresponds to a colored droplet passing through the flow cell (low light transmission due to absorption) and each peak corresponds to a transparent oil segment (high light transmission). The large responses where transmitted light drops to ∼200 units correspond to scattering by the air introduced between samples. The air bubbles applied no adverse effect to the subsequent measurements, which is a distinct feature of the peristaltic pumping system used here and contrasts with syringe pumping, where bubbles can get trapped. An algorithm was used to automatically identify the value of the signal plateau for each oil segment and droplet, and these values were subsequently used to calculate the absorbance of each droplet using a modified version of the Beer–Lambert law:^8^

$$A=-\log_{10}\left(\frac{I_{sample\;droplet}}{I_{blank\;droplet}}\times \frac{I_{blank\;oil}}{I_{droplet\;oil}}\right)=\varepsilon cl$$

where *A* is the absorbance, *I*_sample droplet_ and *I*_blank droplet_ denote the photodiode response due to transmitted light through the sample and blank droplets respectively, which were introduced at the beginning of the experiment. *I*_blank oil_ and *I*_droplet oil_ are the photodiode responses to light transmitted through the carrier fluid next to the sample and blank droplets. To compensate for any small changes in the LED light, the equation includes a component for light transmitted through the carrier fluid.

**Figure 3:**
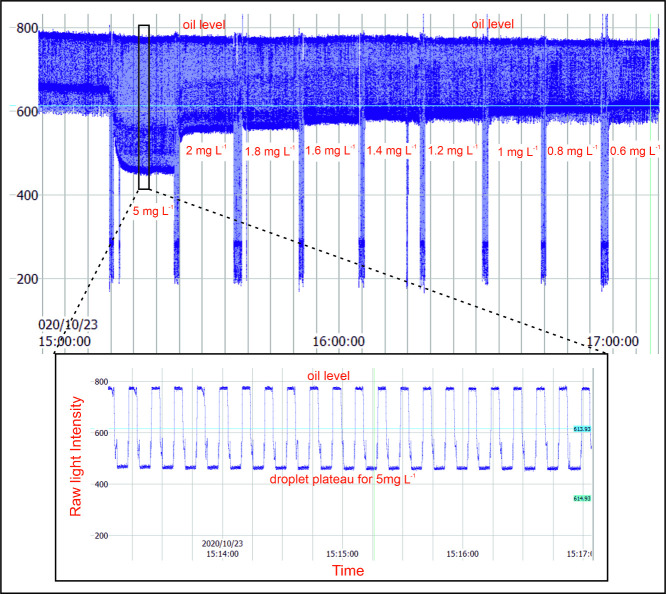
Screenshot from the GUI displaying raw intensity data obtained for calibrating the sensor before deployment. The raw light intensity signal shows the oil levels, and the droplet plateaus correspond to different concentrations of ammonium chloride standard solutions (0.6–5 mg L^–1^). The inset shows a zoomed-in plot of droplet plateau obtained for 5 mg L^–1^ standard solution (duration of about 4 min).

The calibration curves from the benchtop method (Figure 4a) and the droplet microfluidic analyzer (Figure 4b, average of 50 droplets) were compared. Both plots had a high coefficient of correlation (*R* ^2^ > 0.99) of linear regression, with very low SD values (SD < 0.07 mg L^–1^) showing good precision of the results. The absorbance values of the droplet system were about 20 times lower than those from the benchtop spectrophotometer due to the shorter optical path length in the microfluidic flow cell (<0.7 mm versus 10 mm in the spectrophotometer). The LOD was equal to 0.11 mg L^–1^ for the droplet-based analyzer.

**Figure 4:**
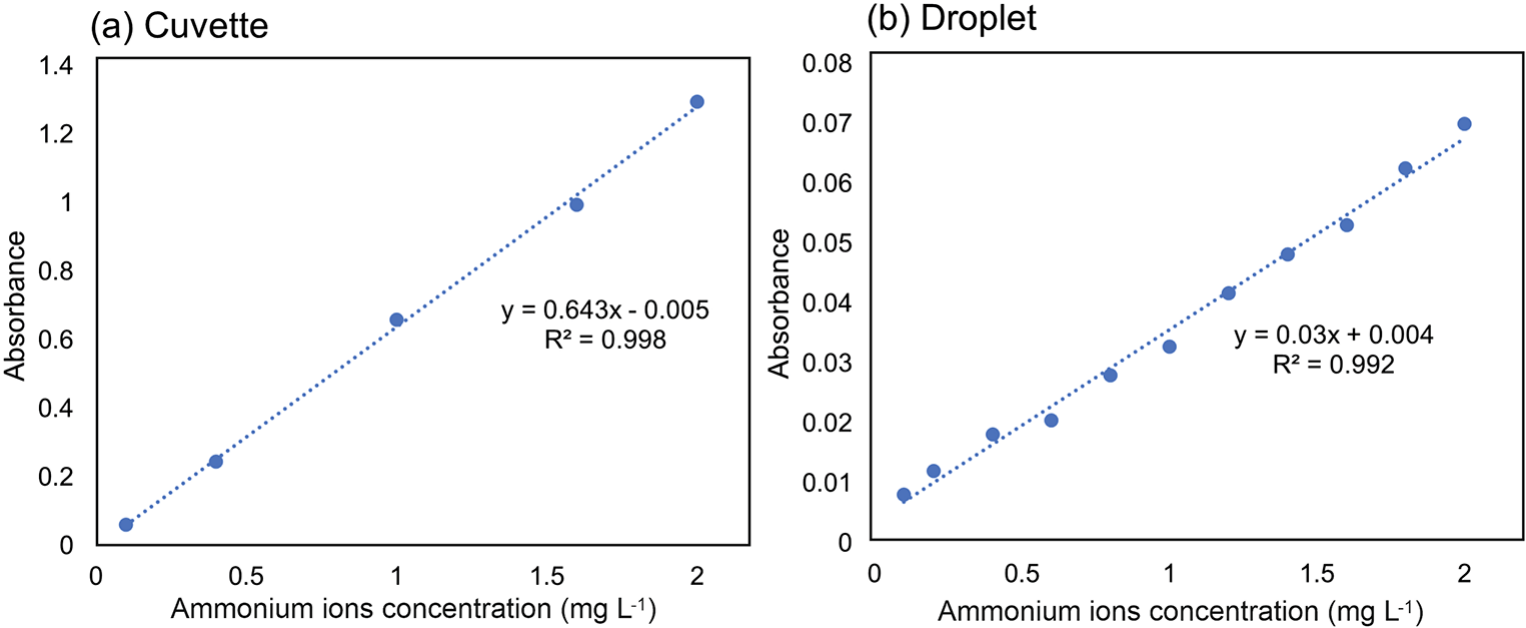
Comparison of results obtained from the benchtop method (a) and the automated droplet-microfluidic system (b). The calibration curves showed linear trends in tested concentration ranges of up to 2 mg L^–1^.

Having demonstrated a linear calibration and determined the droplet microfluidic analyzer’s LOD, we then tested it by measuring water samples taken from the River Itchen (Figure 5a, b). Of the five samples, four were taken from the tidal stretch, with input from a local sewage treatment works. NH_4_^+^ concentrations determined by both methods agreed very well (Figure 5c), with no significant differences (*p* < 0.05). NH_4_^+^ concentrations were higher downstream of the tidal barrier (points 2–5) with results up to 0.4 mg L^–1^. The higher results found at points 3–5 are at similar levels to those previously observed at these locations and were likely due to input from the sewage treatment works outflow located near point 3.^19^ An unexpected observation, however, was that sample 5 had a cloudy appearance following reagent addition in both the benchtop method and the droplet system.

**Figure 5:**
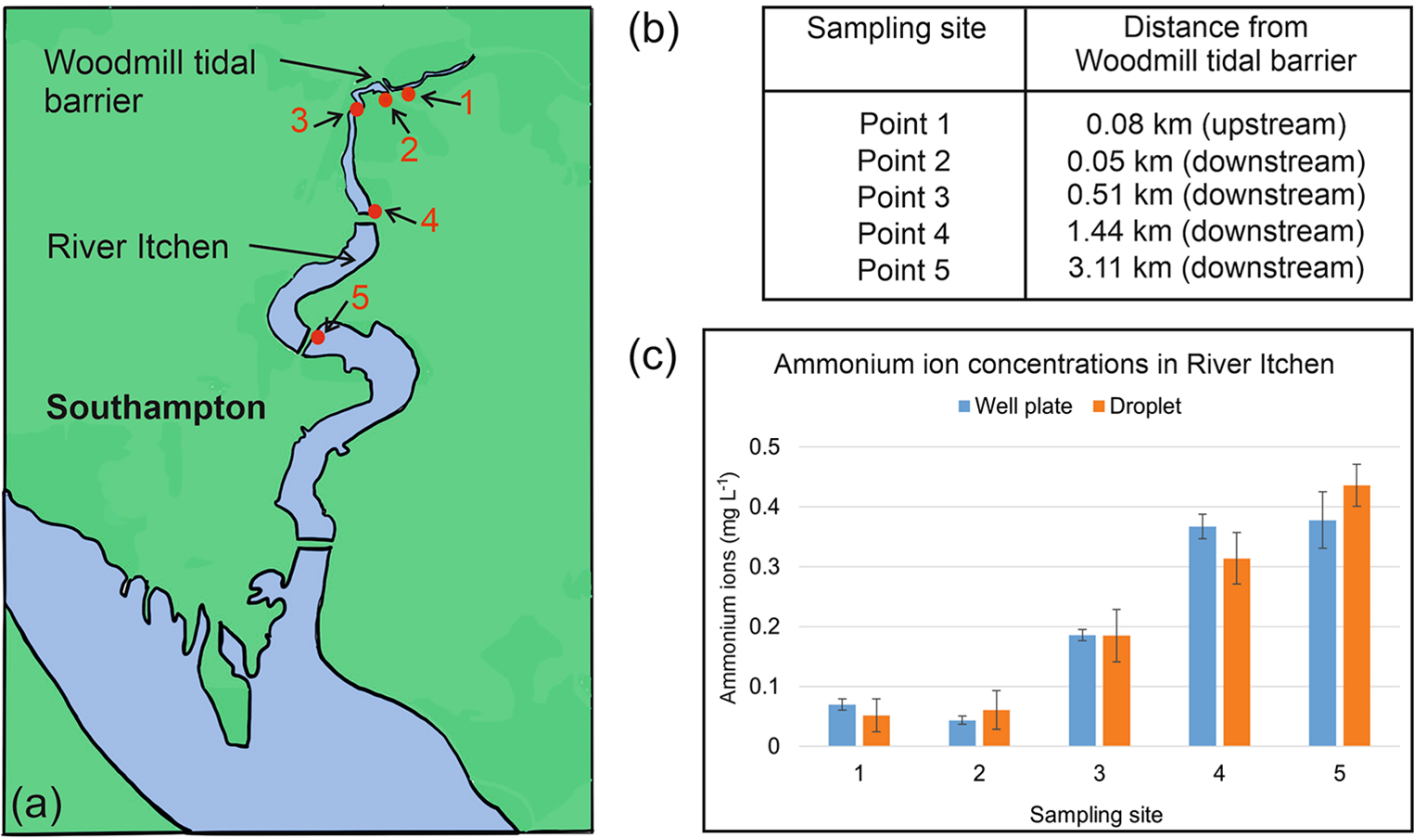
(a) Map showing River Itchen (Southampton, South England) water samples collected from the labeled points (1–5). (b) Distances of sampling points have been tabulated as upstream or downstream from Woodmill tidal barrier on River Itchen. (c) Concentrations of ammonium ions (mg L^–1^) with associated standard error bars in River Itchen samples obtained using well microplate assay (*n* = 3) and droplet analyzer (*n* = 10).

To elucidate this phenomenon, the absorption spectra of both the standards and river water samples were studied after reagent addition (Figure 6), as well as the kinetics of the response at the assay’s characteristic peak (at 660 nm) over time (Figure 7a and 7b). The spectra showed a clear differentiation between sample 5 and all other samples and standards. Samples 1–4 and the standards all showed a characteristic spectral shape with a single peak response centered at 660 nm (Figure 6), with the kinetics indicating that the evolution of that peak had concluded within approximately 6 min (Figure 7). The absorbance spectra of sample 5, however, did not show the characteristic absorption peak at 660 nm, instead a broad response increasing at shorter wavelengths, consistent with scattering caused by precipitate formation. The kinetic profile is also notably different, with the signal continuously increasing in contrast to all other measurements, which plateaued (Figure 7b). As sampling point 5 was furthest downstream and hence would have the highest salinity, the formation of the cloudy precipitate is likely to be linked to the higher salinity, and salt content has previously been shown to cause reagent precipitation.^16^ These results indicate that the assay used in the droplet analyzer have a defined operational range for salinity and would not be valid for NH_4_^+^ monitoring in marine waters.

**Figure 6:**
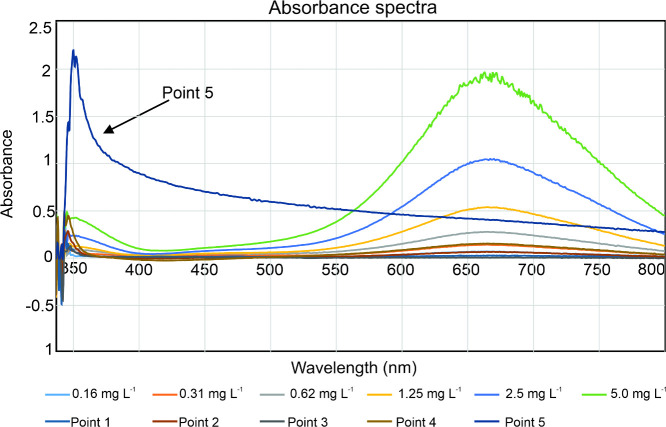
Absorbance spectra (350–800 nm) obtained for six ammonium ions standard solutions (0.16–5.0 mg L^–1^) and five river water samples (point 1–5). All standards and samples showed characteristic spectral profiles with an absorbance maximum at 660 nm, except a river water sample (point 5) which showed a precipitate formation.

**Figure 7:**
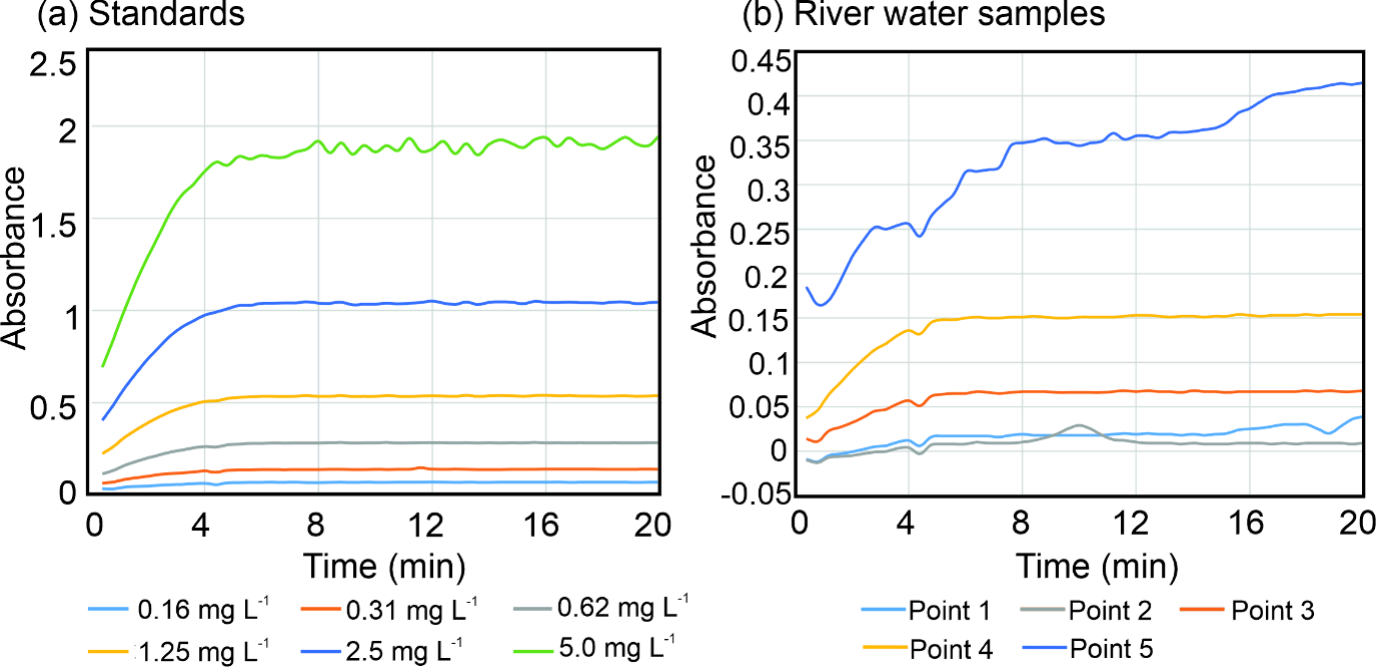
Reaction kinetics of standards (a) and river water samples (b) for measuring ammonium ions. All the standards (a) and river water sample (b) show full color development by approximately 6 min except a river water sample (point 5) which showed precipitate formation.

The prototype system has demonstrated an LOD of 0.11 mg L^–1^ and has been shown to give a linear response up to at least 2 mg L^–1^. Compared to previously reported autonomous wet chemical sensors, the LOD is higher than the best previously reported system^20^ due to the shorter optical path length; however, it could be increased using an improved flow cell with an increased path length^21–23^ or by further optimizing the assay (e.g., improving sample to reagent ratio). Nonetheless, the LOD is broadly consistent if not better than a large number of recently reported analytical field equipment.^24–28^ The measurement range is appropriate for monitoring of NH_4_^+^ in river waters and where similar maximum concentrations have been achieved.^25,28^ In our system, an integrated peristaltic pump was used, which is robust, easy to control, and capable of producing droplets with a low coefficient of variance (<2%), whereas in the other work,^32^ five modified peristaltic pumps were used, which require more time to manufacture and operate the system. A system based on the operation of several pumps simultaneously can introduce more uncertainty into the mixing, which can result in inefficiency and less reliability in the results obtained.

While the system described here was tested on discrete samples, it could be applied to continuous monitoring of different concentration levels of NH_4_^+^ in river water samples. The high measurement frequency (10 s) combined with low reagent consumption (0.3 μL per measurement) means that NH_4_^+^ measurements can be monitored in high detail in near-real-time, over extended durations, without compromising deployment longevity. The high frequency is significantly different than in previously described microfluidic sensors, where measurement times were typically shorter, on the order of several minutes.^24,28^ The high frequency in our system allows for a reduction in instrumental noise, thereby reducing detection limits as well as the possibility of using the sensor on mobile profiling platforms. Despite the high measurement frequency, the overall reagent consumption was significantly lower than that of previously reported systems with microfluidic NH_4_^+^ sensors.^24,25,27^ The oil used in droplet formation was continuously recycled and, therefore, not consumed. This low fluid consumption is important considering the power, length, and frequency of the sensor application. In a recently published work, a droplet-based flow analyzer was developed for the detection of NH_4_^+^ in water using the fluorescent method.^32^ Fluorescent sensing of NH_4_^+^ was performed with *o*-phthalaldehyde (OPA) and sodium sulfite in an alkaline medium, which produces a less stable product that is significantly affected by the pH value. Namely, at pH > 10.4 precipitation of the product can easily occur due to the presence of metal ions in the water samples, which makes fluorescence a more vulnerable detection method compared to the optimized spectrophotometric detection described here. While we have employed an established colorimetric assay, it is evident that the modified Berthelot assay is susceptible to interferences resulting from high salt concentration levels, but which can be improved in future work. We note that previous systems have looked to reduce interferences in NH_4_^+^ measurement by implementing gas diffusion^29,30^ whereby NH_3_ is generated by raising sample pH, then transported through to a clean acceptor fluid where it can be analyzed free from interference. In a droplet system, this could be done by passing between droplets, an effect we have observed in previous work.^31^

The developed automated microfluidic-based system offers promising technology for portable in-line detection of NH_4_^+^, but it is not appropriate for marine environments. Similarly, a low-maintenance NH_4_^+^ monitoring system has been described elsewhere,^33^ addressing the limitations of seawater applications. Recently, a new family of miniaturized lab-on-chip analysers has been reported for the *in situ* monitoring of another N-species, i.e., nitrates.^34^ Unlike nitrates, the detection of NH_4_^+^ in seawater or estuarine environments is more complex^35^ and remains a challenge for newly developed automated microfluidic-based devices.

## 4 Conclusions

In summary, this paper reports the design, calibration, and deployment of an *in situ* droplet microfluidic-based analyzer for continuous monitoring of NH_4_^+^ in natural waters. The analyzer implements the gold-standard colorimetric (modified Berthelot) wet chemistry assay using an integrated pump, heater, optical detector, and other electronic control units, into a self-contained autonomous system. It demonstrated a LOD of 0.11 mg L^–1^ and was shown to provide a linear response up to at least 2 mg L^–1^ for NH_4_^+^ measurements. The microfluidic analyzer can produce droplets with a very low coefficient of variance (<2%), has low reagent consumption (0.3 μL per measurement), and can monitor ammonium ion levels in water with a high measurement frequency (10 s). The system has been successfully validated with samples collected from the River Itchen (Southampton, UK) and has demonstrated great agreement with benchtop spectroscopy results. Therefore, this system can be used for successful and sustainable management of natural waters in near real time.

## Supplementary Material



## Notes

Peristaltic pump components, 3D printed microfluidic chip, and 3D CAD model of the flow cell (PDF)
