# Supplementary material for: Automated Droplet-Based Microfluidic Analyzer for In Situ Monitoring of Ammonium Ions in River Water
Source: ACS ES T Water. 2025 Jul 14;5(8):4387–94. doi: 10.1021/acsestwater.4c01231 (PMC12340750; doi:10.1021/acsestwater.4c01231)
Supplement: Supplementary file 1 [file ew4c01231_si_001.pdf]

Supplementary Information

Automated droplet-based microfluidic analyser for *in-situ* monitoring of ammonium ions in river water

Wahida T. Bhuiyan[a], Jelena Milinovic[a], Brett Warren[b], Yong-Qiang Liu[b], Adrian M. Nightingale[a], and Xize Niu[a*]

[a] Faculty of Engineering and Physical Sciences, University of Southampton, Southampton, SO17 1BJ, UK

[b] SouthWestSensor Ltd, 2 Venture Rd, Chilworth, Southampton SO16 7NP, UK

* Email: x.niu@soton.ac.uk

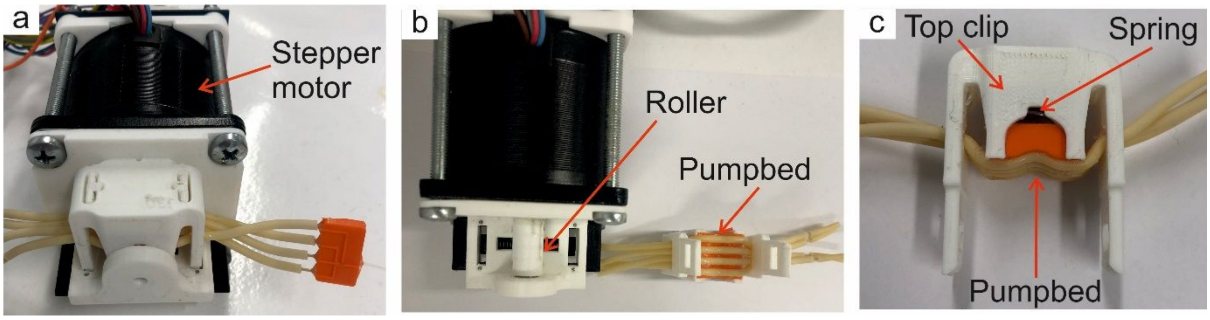

Figure S1. Peristaltic pump components built in-house. (a) A stepper motor of the pump, 3D printed pump head and four pump lines. An orange microfluidic chip connected to the pump lines. (b) Inside view of the pump head showing a 3D printed roller with features and pumpbed. (c) Side view of the top clip of the pump head, spring, and pump bed.

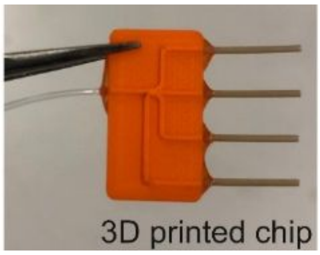

Figure S2. 3D printed microfluidic chip showing four inlets and an outlet.

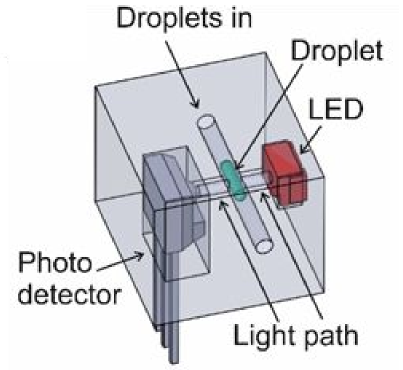

Figure S3. 3D CAD model of the flow cell showing the flow path and grooves for securing the photodiode and LED.
